# Effects of aerobic training with blood flow restriction on aerobic capacity, muscle strength, and hypertrophy in young adults: a systematic review and meta-analysis

**DOI:** 10.3389/fphys.2024.1506386

**Published:** 2025-01-07

**Authors:** Zhendong Gao, Yan Li, Jinjin Zhang, Liqiang Li, Tao Wang, Xiaolin Wang, Hao Wang

**Affiliations:** ^1^ Faculty of Educational Studies, University Putra Malaysia, Selangor, Malaysia; ^2^ Department of Sports Teaching and Research, Lanzhou University, Lanzhou, China; ^3^ School of Physical Education, Xi’an Peihua University, Xi’an, China; ^4^ School of Physical Education, Xizang Minzu University, Xianyang, China; ^5^ School of Physical Education, Shenyang Medical College, Shenyang, China

**Keywords:** blood flow restriction, aerobic training, aerobic capacity, muscle strength, muscle hypertrophy

## Abstract

Aerobic training with blood flow restriction (AT-BFR) has shown promise in enhancing both aerobic capacity and exercise performance. The aim of this review was to systematically analyze the evidence regarding the effectiveness of this novel training method on aerobic capacity, muscle strength, and hypertrophy in young adults. Studies were identified through a search of databases including PubMed, Scopus, Web of Science, SPORTDiscus, CINAHL, Cochrane Library, and EMBASE. A total of 16 studies, involving 270 subjects, were included in the meta-analysis. The results revealed that AT-BFR induced greater improvements in VO_2max_ (SMD = 0.27, 95%CI: [0.02, 0.52], *p* < 0.05), and muscle strength (SMD = 0.39, 95%CI: [0.09, 0.69], *p* < 0.05), compared to aerobic training with no blood flow restriction (AT-noBFR). However, no significant effect was observed on muscle mass (SMD = 0.23, 95%CI: [-0.09, 0.56], *p* = 0.162). Furthermore, no moderating effects on the outcomes were found for individual characteristics or training factors. In conclusion, AT-BFR is more effective than AT-noBFR in improving aerobic capacity and muscle strength, making it a promising alternative to high-intensity training.

**Systematic Review Registration:**

https://www.crd.york.ac.uk/prospero/, identifier CRD42024559872.

## Introduction

Aerobic exercise offers significant benefits for athletic performance and overall health, including improved cardiorespiratory fitness (Andreu-Caravaca et al., 2021; Huang et al., 2005), improved muscle quality (Lee and Stone, 2020; Markov et al., 2022), enhanced recovery (Paneroni et al., 2017), and reduced cardiovascular disease risk (Brouwer et al., 2021; Kodama et al., 2009). However, low-intensity aerobic exercise may not be sufficient to achieve substantial improvements in these aspects. The American College of Sports Medicine recommends 5–7 days of moderate-intensity aerobic exercise or 3 days of vigorous exercise per week for adults to improve health and prevent disease (Garber et al., 2011). As aerobic capacity and performance improve, higher-intensity aerobic fitness methods are required to elicit training adaptations (Medicine, 2013). Nonetheless, high-intensity training is not suitable for some specific populations, such as the elderly, patients undergoing rehabilitation, or in-season athletes. Therefore, developing a low-intensity training method that can achieve similar benefits to high-intensity training is of great significance.

Blood flow restriction (BFR) training has gained increasing popularity in the fields of sports and rehabilitation (Hughes et al., 2017; Loenneke et al., 2010). This method involves applying an external constricting device to the proximal limbs to partially restrict venous return, thereby creating a hypoxic and stressful environment that promotes physical adaptations (Jessee et al., 2018). Previous meta-analyses have found that BFR resistance training can achieve effects similar to high-intensity resistance exercise, specifically regarding muscle strength and hypertrophy, while minimizing mechanical load (20–30% one repetition maximum) (Centner et al., 2019; Grønfeldt et al., 2020; Lixandrão et al., 2018). This suggests that BFR training may provide a viable alternative to high-intensity resistance training for the development of muscular strength and hypertrophy.

In effect, AT-BFR may offer an effective and practical method to improve aerobic capacity, muscle strength, and hypertrophy, particularly in older adults, clinical rehabilitation populations, and athletes during periods of reduced training intensity. Despite its potential benefits for these groups, most studies on AT-BFR have focused on young adults, primarily due to their better health and greater training adaptability, which help control experimental conditions and minimize confounding factors (Formiga et al., 2020; Silva et al., 2019). Recent research has aimed to determine the effects of AT-BFR on aerobic capacity and muscle performance in young adults, yielding conflicting results (Chen et al., 2021; Herda et al., 2024; Keramidas et al., 2012). While a previous meta-analysis has been conducted, it exhibits notable limitations. The meta-analysis by Formiga et al. (2020) focused exclusively on aerobic capacity, while the meta-analysis by de Lemos Muller et al. (2024) examined only muscle strength and hypertrophy, with both including a limited number of studies. Additionally, the review by Bennett and Slattery (2019) explored the effects of aerobic BFR on aerobic capacity and performance but relied on a narrative review without meta-analytical rigor, limiting the quantitative synthesis of evidence. These studies, while valuable, fail to comprehensively assess AT-BFR’s combined effects on aerobic capacity, muscle strength, and hypertrophy. Furthermore, individual characteristics (e.g., sex, training status) and training variables (e.g., intensity, frequency) are likely to moderate these outcomes (Wang et al., 2023a; Wilk et al., 2018), which highlights the need to evaluate their potential influences to better understand AT-BFR’s overall effectiveness. Investigating these outcomes together is crucial, as they represent the multidimensional adaptations influenced by AT-BFR. By creating localized hypoxia, increasing metabolic stress, and altering muscle fiber recruitment, AT-BFR triggers adaptations in both aerobic and muscular systems, making it effective for enhancing overall physical performance (Pope et al., 2013; Smith et al., 2022). These adaptations are essential for designing training protocols that balance cardiovascular and muscular performance.

This study aims to systematically evaluate the effects of AT-BFR on aerobic capacity, muscle strength, and hypertrophy in young adults, as well as well as the moderating effects of individual characteristics (e.g., gender, training level) and training variables (e.g., training duration, frequency, intensity, cuff pressure) on training outcomes. The findings could help inform the design of more personalized and optimized training protocols and improve the applicability of AT-BFR in both athletic and clinical settings.

## Materials and methods

### Search strategy and study selection

**FIGURE 1:**
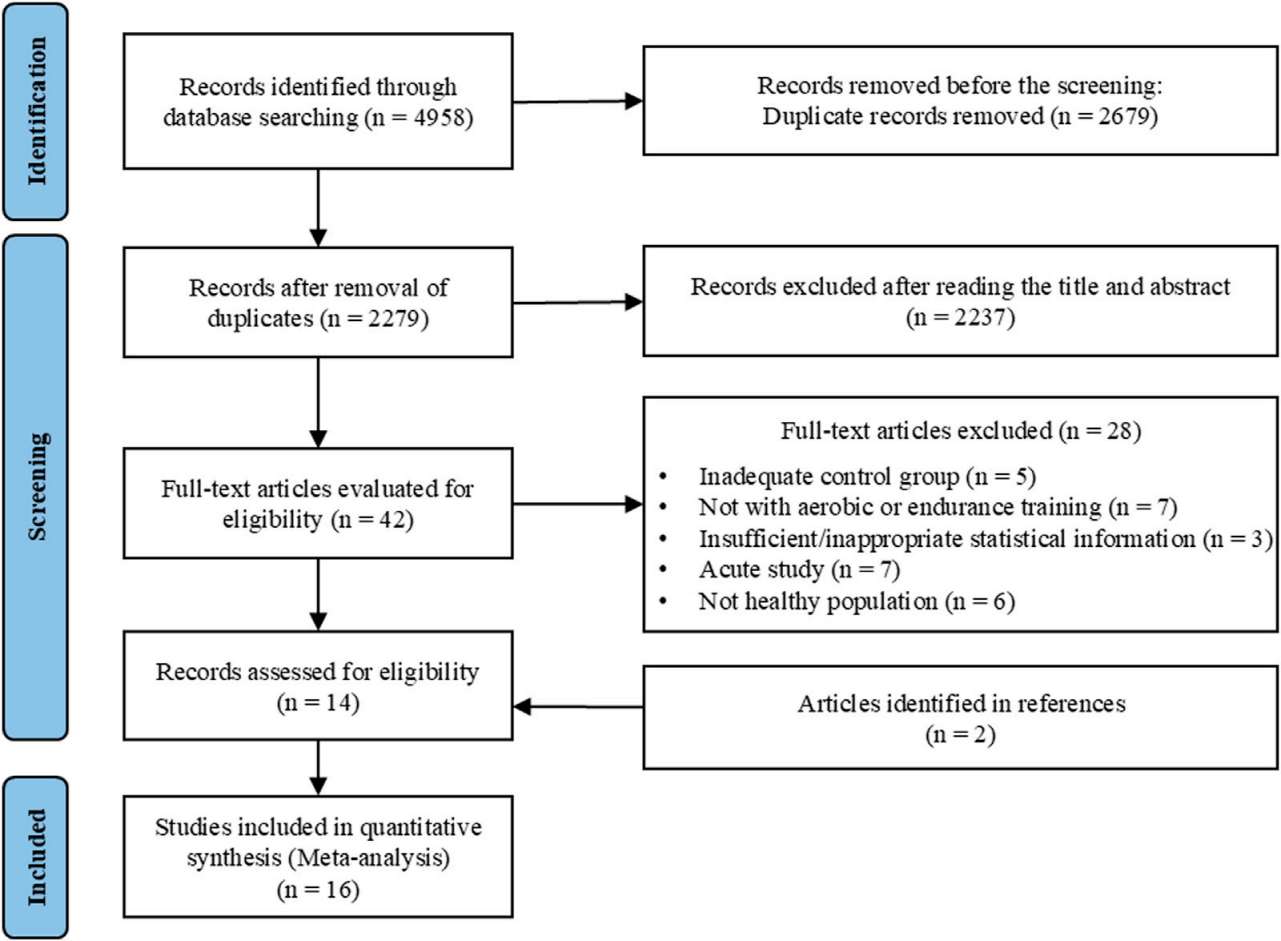
PRISMA flow diagram.

This systematic review and meta-analysis were conducted in accordance with the PRISMA guidelines (Page et al., 2021) (Prospero registration number: CRD42024559872). Comprehensive searches were conducted across multiple electronic databases, including PubMed, Scopus, Web of Science, SPORTDiscus, CINAHL, Cochrane Library, and EMBASE, up to 15 June 2024. Boolean operators AND and OR were applied to predefined combinations of keywords and MeSH terms in each database: (“blood flow restriction therapy” OR “ischemia” OR “vascular occlusion” OR “tourniquets” OR “occlusion training”) AND (“endurance exercise” OR “aerobic exercise” OR “cycling” OR “running” OR “walking”). Detailed search strings are provided in Supplementary Material 1. After deduplication, the titles and abstracts of the retrieved articles were screened, followed by a full-text review (Figure 1). Additionally, reference lists of included studies were scrutinized for further relevant articles. Two researchers (T. W. and X.W.) independently retrieved articles, with any discrepancies resolved by a third researcher (Z.G.).

### Eligibility criteria

Eligibility criteria for article inclusion were as follows: (a) healthy young adults; (b) the study design allowed comparisons between AT-BFR and AT-noBFR; (c) aerobic capacity, muscle strength and/or muscle hypertrophy were assessed pre- and post-training; (e) publications in English.

### Methodological quality assessment and risk of bias

The quality of the included studies was assessed using the PEDro scale (Verhagen et al., 1998), which evaluates methodologies based on 11 criteria, such as randomization, blinding, and outcome measures. The scale has a maximum score of 10 points, with the first item not being scored. Following established research standards (Stojanović et al., 2017; Wang et al., 2024), studies scoring below 4 on the PEDro scale were considered low quality. In addition, the revised Cochrane risk-of bias tool for randomized trials (RoB-2 tool version 2) was employed to assess potential bias across five domains: randomization, deviations from the intended intervention, missing data, outcome measurement, and selective reporting (Sterne et al., 2019). The assessment of methodological quality was independently conducted by two reviewers (Y.L. and J.Z.), with any discrepancies resolved by consensus with a third reviewer (L. L.). Additionally, potential bias was evaluated through visual inspection of funnel plots and Egger’s test.

### Data extraction

The following data were extracted: participant demographics (i.e., age, gender, training status), and study characteristics (i.e., training duration, frequency, intensity, volume, occlusion pressure). For outcome measures, data were collected on aerobic capacity testing (e.g., VO_2max_ or lactate threshold), muscle strength testing (e.g., dynamic, isometric, and isokinetic testing), and muscle mass testing (e.g., assessed by magnetic resonance imaging, and ultrasound). When multiple time points for training outcomes were available, the latest time point was used as the post-training value for analysis. In cases where the required data were unavailable, they were requested directly from the authors. In the absence of a response, the study outcome was excluded. The specific characteristics of the participants and plyometric training protocol were presented in Table 1.

**TABLE 1:** Characteristics of the included studies.

Author	Subjects	Protocol and N	Exercise mode	Cuff pressure	Duration; frequency	Outcomes (percentage increase)
Abe et al. (2006)	healthy young men (21.4 ± 2.8 years)	BFR, 9 CG, 9	5 × 2-min treadmill walking (50 m/min)	160–230 mmHg	4 weeks 6 days/wk	Muscle mass (quadriceps, hamstrings, adductors): BFR, 4.1%–7.6%, CG, −1.7%−1.5% Maximal strength (leg press and curl): BFR, 7.4%–8.3%, CG, −2.9%−1.9%
Abe et al. (2010)	healthy young men (23.0 ± 1.7 years)	BFR, 11 CG, 8	BFR:15min cycling (40% VO_2max_); CG: 45 min cycling (40% VO_2max_)	160–210 mmHg	8 weeks 3 days/wk	Muscle mass (thigh and quadriceps): BFR, 3.8%–5.1%, CG, −1% Maximal strength (knee extension and flexion): BFR, 3.3%–7.7%, CG, −3.4%−1.4% VO_2max_: BFR, 5.8%, CG, 0.5%
Amani et al. (2018)	Male soccer players (23.9 ± 2.3 years)	BFR, 10 CG, 9	3–4 × 400 m running (60–70% HRR)	NG	2 weeks 4 days/wk	VO_2max_: BFR, 3.7%, CG, 1.5%
Beak et al. (2022)	Male runners (30 ± 4.1 y)	BFR, 14 CG, 15	5 × 2 min treadmill running (40% VO_2max_)	160–240 mmHg	8 weeks 3 days/wk	Muscle mass (thigh): BFR, 1.4%, CG, −1.5% VO_2max_: BFR, 6.4%, CG, 5.7% Jump power: BFR, 8.9%, CG, 8.6%
Chen et al. (2021)	Endurance male athletes (21.6 ± 0.8 years)	BFR, 10 CG, 10	4 × 3 min treadmill running (50% HRR)	154 ± 6 mmHg	8 weeks 3 days/wk	Muscle mass (trunk, right leg, left leg): BFR, 4.8%, CG, 1.3% Maximal strength (knee extension and flexion): BFR, 7.1%–13%, CG, 0–7.7%
Chen et al. (2022)	Endurance male athletes (21.6 ± 2.2 years)	BFR, 10 CG, 10	5 × 3 min treadmill running (50% HRR)	154 ± 6 mmHg	8 weeks 3 days/wk	Maximal strength (knee extension and flexion): BFR, 9.4%–9.9%, CG, 1.5%–2.4% Maximal running performance: BFR, 12.6%, CG, 4.5% Muscle endurance: BFR, 8.9%–9.1%, CG, −2%−7%
Conceicao et al. (2019)	healthy young men (23.5 ± 2.6 years)	BFR, 10 CG, 10	30 min cycling (40% VO_2max_; 70% VO_2max_)	95 ± 4 mmHg	8 weeks 4 days/wk	Muscle mass (femur): BFR, 10.7%, CG, 3.8% Maximal strength (leg press): BFR, 9%, CG, 3% VO_2max_: BFR, 11%, CG, 21%
de Oliveira et al. (2016)	healthy young adults (23.8 ± 4 years)	BFR, 10 CG, 7	5–8 × 2 min cycling (30% VO_2max_)	140–200 mmHg	4 weeks 3 days/wk	Maximal strength (knee extension): BFR, 11.4% ± 7.3%, CG, −2.6% ± 6.7% VO_2max_: BFR, 5.6% ± 4.2%, CG, 0.4% ± 4.7%
Held et al. (2020)	Elite rowers (21.8 ± 3.5 years)	BFR, 16 CG, 15	2 × 10 min endurance rowing (65% HRR)	75% max length	5 weeks 3 days/wk	Maximal strength (squat): BFR, 5.4% ± 5.7%, CG, 4.6% ± 5.3% VO_2max_: BFR, 9.1% ± 6.2%, CG, 2.5% ± 6.1%
Held et al. (2023)	Male swimmers (22.7 ± 3 years)	BFR, 10 CG, 8	Low-intensity swimming	135 ± 10 mmHg	5 weeks 3 days/wk	VO_2max_: BFR, 2.5%, CG, −4.7% Swimming speed at first lactate threshold: BFR, −0.9%, CG, 0.9% Swimming speed at second lactate threshold: BFR, 0, CG, 0.8%
Herda et al. (2024)	Highly trained runners (32.9 ± 11 years)	BFR, 11 CG, 11	10-min walking. (4.83 km/h)	NG	4 weeks 3 days/wk	Skeletal muscle mass: BFR, −0.9%, CG, 1.4% VO_2max_: BFR, 2.7%, CG, 2.1% Time to exhaustion: BFR, 5.2%, CG, 7.6%
Keramidas et al. (2012)	Trained young adults (23 ± 4.3 years)	BFR, 10 CG, 10	2 min of cycling (90% VO_2max_)	90 mmHg	6 weeks 3 days/wk	VO_2max_: BFR, −2.2%, CG, −4.2%
Kim et al. (2016)	Healthy young males (22.4 ± 3.0 years)	BFR, 11 CG, 10	20-min cycling at 30% HRR	160–180 mmHg	6 weeks 3 days/wk	Muscle mass (thigh): BFR, 2.5% CG, 1.4% Maximal strength (knee extension and flexion): , BFR, 6–7.1%, CG, 1.3–3.6% VO_2max_: BFR, 2%, CG, −1.2%
Park et al. (2010)	Male basketball players (20.4 ± 1.2 years)	BFR, 7 CG, 5	5 × 3-min walking (4–6 km/h, 40% VO_2max_)	160–230 mmHg	2 weeks 6 days/wk	Maximal strength (knee extension and flexion): BFR, 3.7%–20.4%, CG, −1.2−13.6% VO_2max_: BFR, 11.5%, CG, −1.3%
Paton et al. (2017)	Health active adults (24.9 ± 6.9 years)	BFR, 8 CG, 8	10–24 × 2 min treadmill running (80% HRR)	NG	4 weeks 2 days/wk	VO_2max_: BFR, 6.3%, CG, 3.9% Running economy: BFR, 6.7%, CG, −2.1% Time to exhaustion: BFR, 25.8%, CG, 6.6%
Thompson et al. (2024)	Recreationally active adults (26 ± 11 years)	BFR, 8 CG, 10	5 × 3-min walking (5 km/h, 5% grade)	100% LOP	4 weeks 2 days/wk	VO_2max_: BFR, 9–9.1%, CG, 1.3–1.6%

Note: BFR, blood flow restriction training; CG, control group; HRR, heart rate reserve; LOP, lowest occlusion pressure; NG, not given; wk, week/s; y, years.

### Statistical analyses

All meta-analyses were conducted using R version 4.3.0 (R Foundation for Statistical Computing, Vienna, Austria). Meta-analysis was conducted using the *metacont()* function from the *meta* package, with subgroup analyses performed via the *update()* function. Sensitivity analyses were carried out using the *InfluenceAnalysis()* function from the *dmetar* package. The effect size difference for between-group comparisons (AT-BFR vs. AT-noBFR) was calculated using pre- and post-intervention data (mean, standard deviation, and sample size). The change in standard deviation ( ${SD}_{change}$ ) was determined using the following equation:

$${SD}_{change}=\sqrt{\left({{SD}_{pre}}^{2}/N_{pre}\right)+\left({{SD}_{post}}^{2}/N_{post}\right)}$$

The magnitude of effect size was categorized as follows: <0.40 = small, 0.40–0.70 = moderate, and >0.70 = large (Higgins, 2008). A random effects model was employed to account for heterogeneity and measurement variability among the included studies. Heterogeneity was verified with the *I* ^2^ statistic, with *I* ^2^ ≤ 25% indicating low heterogeneity, 25%–75% indicating moderate heterogeneity, and >75% indicating high heterogeneity (Higgins et al., 2003).

A total of three meta-analyses were conducted. The analyses examined the impact of AT-BFR versus AT-noBFR on aerobic capacity (VO_2max_), maximal muscle strength, and muscle mass, which were the primary outcomes of this systematic review and meta-analysis. Additionally, Secondary outcomes were evaluated through subgroup analyses, which examined the potential moderating effects of variables such as gender, training status (trained or untrained), training duration (<8 weeks and ≥8 weeks), training frequency (≤3 days/week and >3 days/week), training intensity (low intensity, walking; moderate intensity, running, swimming, or rowing), occlusion pressure (<180 mmHg or ≥180 mmHg) on these primary outcomes. The threshold for statistical significance was set at *p* < 0.05.

The meta-analysis involved some deviations from the registered protocol. To enhance homogeneity, the analysis focused on a more consistent participant group, specifically younger adults. Furthermore, subgroup analyses were conducted only when each subgroup included at least three homogeneous datasets, resulting in the exclusion of certain subgroups due to a limited number of studies.

## Results

### Study selection

A total of 4,958 studies were identified in the initial search. After title and abstract screening, 42 studies were subjected to full-text review based on the eligibility criteria. Of these, 14 studies met the inclusion criteria. Additionally, two studies were identified through reference lists of the selected articles. Ultimately, 16 studies were included in the final systematic review and meta-analysis. An overview of these studies is provided in Figure 1.

### Methodological quality assessment and risk of bias

The quality assessment of the included studies identified 9 studies as moderate quality (scores of 4–5) and 10 studies as high quality (scores of 6–10). With a median score of 6 out of 10, the overall quality of the studies was determined to be moderate to high, supporting the reliability of the findings. Detailed PEDro scale scores are provided in the Supplementary Material 2.

**FIGURE 2:**
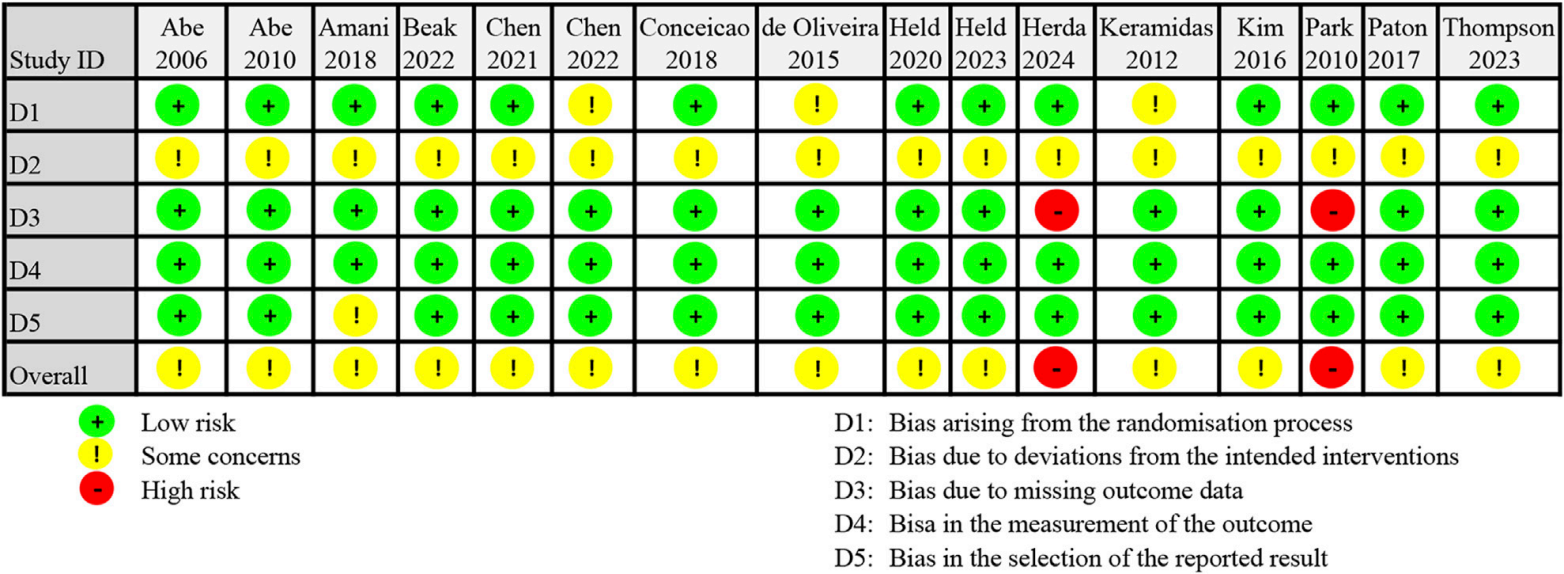
Risk of bias assessment of including trials using Rob2 tool.

The ROB2 assessment revealed that 14 studies were rated as having some concern regarding risk of bias, while other 2 studies were classified as high risk (see Figure 2). All studies exhibited deviations from the intended interventions (Domain 2), attributed to the lack of access to trial protocols and absence of trial registration information. In Domain 1, baseline differences between intervention groups in three studies raised concerns regarding group comparability. Two studies were classified as high risk in Domain 3 due to outcome data being available for fewer than 85% of participants. In Domain 4, all studies were considered low risk for measurement methods, as reliable instruments were employed. One study showed some results that appeared unreasonable, raising concerns in Domain 5. Detailed risk of bias percentages are provided in Supplementary Material 3.

Egger’s test revealed no significant publication bias for VO_2max_ (b = 2.38, t = 1.85, *p* = 0.09), while it was not applicable for muscle strength and muscle mass due to the limited number of studies (n < 10). The funnel plots from the four meta-analyses demonstrated a relatively symmetrical distribution, suggesting no significant publication bias or selective reporting (see Supplementary Material 4).

### Meta-analysis results

**FIGURE 3:**
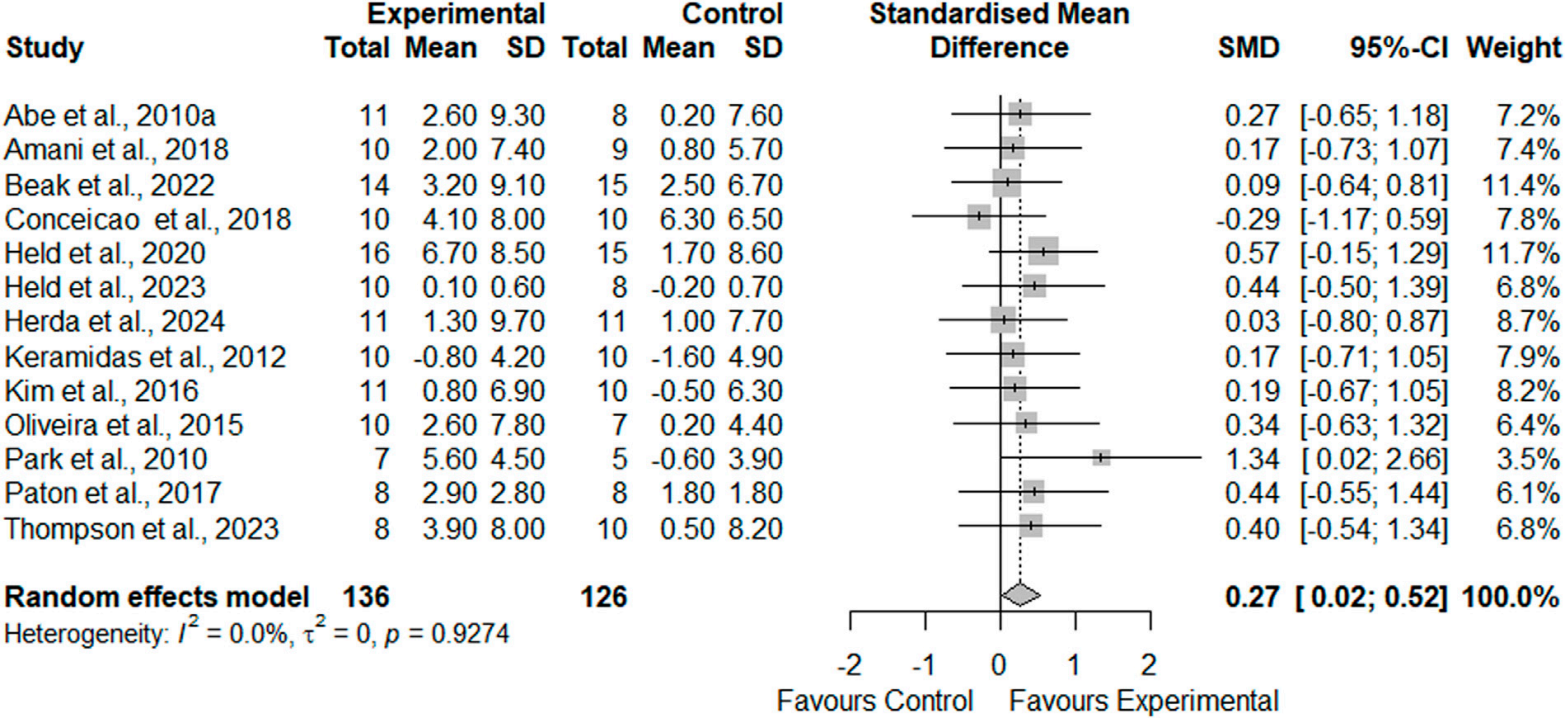
Forest plot demonstrating the effects of aerobic training with blood flow restriction vs. without blood flow restriction on VO_2max_.

Thirteen studies comparing the effects of AT-BFR and AT-noBFR were included in this meta-analysis (see Figure 3). The mean VO_2max_ gain was 5.7% ± 3.2% for the AT-BFR group and 2.4% ± 7.0% for the AT-noBFR group. The meta-analysis results showed AT-BFR had a small effect on VO_2max_ compared to AT-noBFR (SMD = 0.27, 95%CI: [0.02, 0.52], *p* = 0.031 < 0.05). The *I* ^2^ statistic indicated minimal heterogeneity (0%).

**FIGURE 4:**
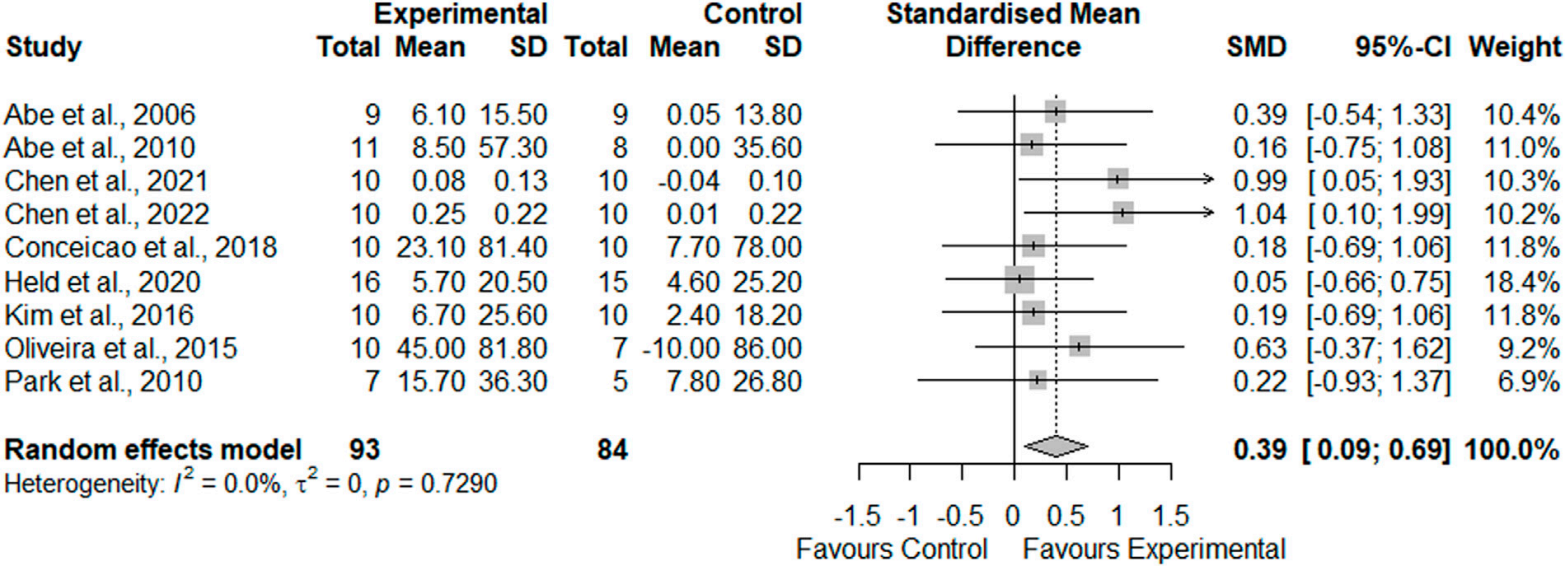
Forest plot demonstrating the effects of aerobic training with blood flow restriction vs. without blood flow restriction on muscle strength.

Nine studies comparing the effects of AT-BFR and AT-noBFR were included for meta-analysis (Figure 4). Across comparisons, AT-BFR resulted in an average percentage increase of 7.3% ± 2.5% in muscle strength, compared to AT-noBFR with 2.3% ± 2.5%. Quantitative analyses demonstrated that AT-BFR had a moderate effect on muscle strength compared to AT-noBFR (SMD = 0.39, 95%CI: [0.09, 0.69], *p* = 0.011 < 0.05). The I^2^ statistic indicated minimal heterogeneity (0%).

**FIGURE 5:**
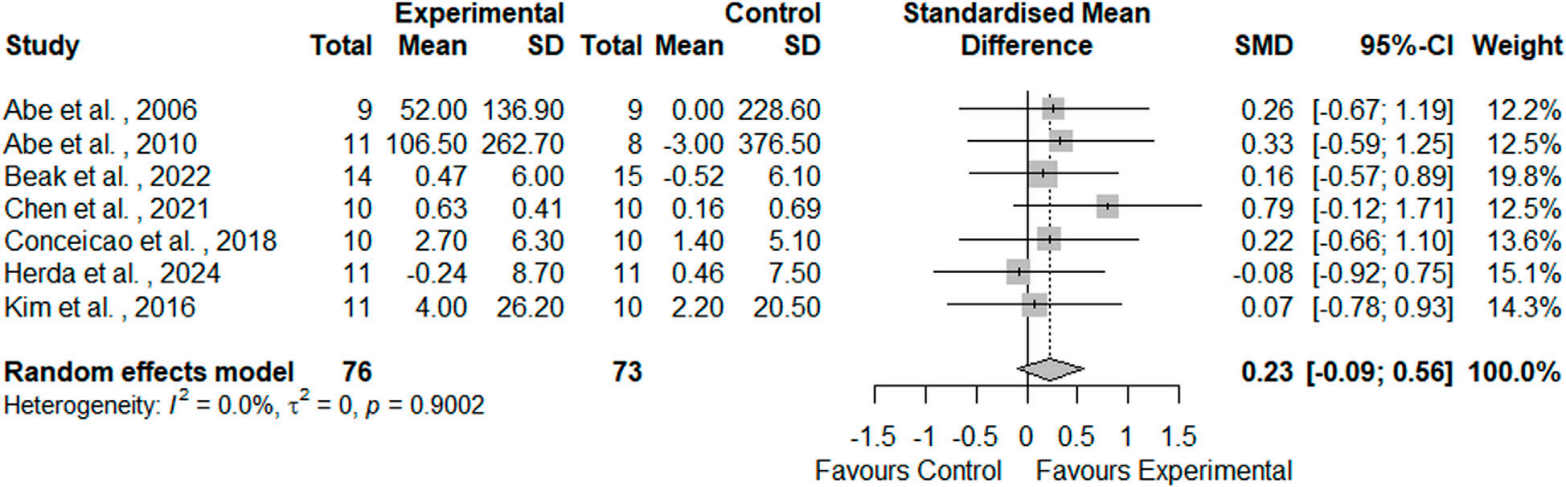
Forest plot demonstrating the effects of aerobic training with blood flow restriction vs. without blood flow restriction on muscle mass.

Seven studies comparing the effects of AT-BFR and AT-noBFR on muscle mass were included in this meta-analysis (Figure 5). Between-group comparisons showed higher increases in muscle mass following AT-BFR (5.8% ± 2.7%) compared to AT-noBFR (2.1% ± 2.2%). Statistical examination revealed that AT-BFR had a small effect on muscle mass compared to AT-noBFR (SMD = 0.23, 95%CI: [-0.09, 0.56], *p* = 0.162). The *I* ^2^ statistic indicated minimal heterogeneity (0%).

The sensitivity analysis revealed no significant changes, with effect sizes and heterogeneity remaining stable after excluding individual studies, further confirming the robustness and reliability of the results (see Supplementary Material 5). Subgroup analyses were conducted if at least three relatively homogeneous datasets were available for each subgroup. A total of 13 subgroup analyses were performed for aerobic capacity, muscle strength, and hypertrophy based on gender, training level, training intensity, training frequency, training duration, and occlusion pressure (see Supplementary Material 6). The results showed that all subgroup analyses were non-significant.

## Discussion

This meta-analysis compared the effects of AT-BFR and AT-noBFR on aerobic capacity (i.e., VO_2max_), muscle strength, and muscle mass. The main findings indicated that AT-BFR induced greater improvements in VO_2max_ and maximal strength compared to AT-noBFR. However, no significant differences were observed in muscle mass. Additionally, personal characteristics and training-related factors did not appear to significantly moderate the training outcomes.

With regard to aerobic capacity, this meta-analysis found that AT-BFR was more effective in improving VO_2max_ than AT-noBFR, consistent with the meta-analysis by Formiga et al. (2020). Furthermore, several studies included in this meta-analysis reported superior improvements in running economy and time to exhaustion with AT-BFR compared to AT-noBFR (Herda et al., 2024; Paton et al., 2017). The enhanced aerobic capacity observed with AT-BFR can be attributed to several potential mechanisms. Firstly, BFR exercise induces higher heart rate and blood pressure during activity, creating increased cardiovascular stress and stimulating adaptive cardiovascular responses (Takano et al., 2005). Secondly, Second, BFR-induced hypoxia, resulting from reduced oxygen delivery and impaired metabolite clearance, triggers increased oxidative stress, activating AMPK signaling pathways that are crucial for mitochondrial biogenesis and cellular energy regulation (Christiansen et al., 2018). These molecular adaptations improve mitochondrial function, angiogenesis, and capillary density within muscle tissue, facilitating better oxygen delivery and utilization (Barjaste et al., 2021; Christiansen et al., 2020). Moreover, the fluid shear stress caused by ischemia and reperfusion during BFR exercise may strongly promote the expression of angiogenesis-related factors, contributing to vascular adaptation (Hudlicka and Brown, 2009). These combined mechanisms likely contribute to the observed improvements in both aerobic capacity.

Although this study did not find a significant effect of AT-BFR on muscle mass, the analysis showed that AT-BFR significantly enhanced maximal strength. This phenomenon can be attributed to the mechanisms of blood flow restriction training, which involves recruitment of fast-twitch fibers, stimulation of protein synthesis, and activation of anabolic growth factors, all of which play a key role in strength improvement (Crane et al., 2013; Loenneke et al., 2010; Markov et al., 2022). However, the lack of significant muscle mass improvement may be due to the limitations of low-intensity aerobic training in promoting muscle hypertrophy. Aerobic training primarily enhances exercise performance through improvements in cardiovascular endurance, with relatively minor effects on muscle mass, especially at lower training intensities (Hendrickse et al., 2021; Jones and Carter, 2000). In contrast, de Lemos Muller (2024) reported increases in muscle hypertrophy, likely due to their use of localized measures such as cross-sectional area, which are more sensitive to site-specific adaptations. Additionally, their inclusion of a broader age range (18–60 years) may have introduced greater variability in baseline muscle characteristics and adaptive potential, compared to our focus on young adults with more uniform responses. These differences highlight the specificity of AT-BFR’s effects and suggest it is more effective for increasing strength than muscle mass, particularly in training programs balancing strength and endurance or in low-intensity regimens for injured athletes.

Although individual and training factors did not significantly moderate training outcomes, this systematic review and meta-analysis provides valuable insights. No significant differences were found between training intensities (40%–90% VO_2max_), indicating that low-intensity (40%–60% VO_2max_) AT-BFR can produce similar effects to high-intensity training. This makes it particularly beneficial for individuals undergoing rehabilitation or athletes who need to maintain performance during the competitive season without overtraining. Regarding training duration, while the studies included in this meta-analysis ranged from 2 to 8 weeks, even short-term (2–4 weeks) training was effective in significantly improving aerobic capacity and strength, making it suitable for athletes requiring rapid recovery or performance maintenance. A training frequency of 2–6 days per week was also effective, demonstrating the flexibility of AT-BFR in various training programs. Regarding cuff pressure, no significant impact on training outcomes was observed within the 90–240 mmHg range. However, for individuals with lower physical capacity or cardiovascular conditions, it is recommended to use lower occlusion pressure to enhance safety. Overall, AT-BFR demonstrates broad adaptability across different training conditions. However, these findings are based on a limited number of studies, and further research is needed to strengthen the evidence base and optimize its application.

### Limitations

This meta-analysis has several limitations that should be carefully considered in the interpretation of the findings. Although BFR training is widely discussed in scientific research (Hughes et al., 2017; Scott et al., 2015; Wang et al., 2023b), the number of studies examining the effects of AT-BFR is still sparse. More high-quality studies are needed in the future to enhance the robustness and applicability of the results. Secondly, while the included studies have not documented adverse reactions or injuries associated with AT-BFR, this does not imply that the training is devoid of potential safety concerns. Thirdly, while most studies were of high quality, some were of fair quality, and nearly all failed to blind participants, coaches, or assessors. Future research should improve blinding and randomization procedures, and provide detailed reports on study design and analysis methods to enhance the reliability and reproducibility of results.

## Conclusion

The present meta-analysis demonstrated that AT-BFR significantly improves VO2max and maximal strength compared to AT-noBFR, with no significant effect on muscle mass. Individual characteristics and training factors did not notably influence these outcomes. AT-BFR appears to provide a viable and effective alternative to high-intensity training.

From a practical standpoint, aerobic exercise plays a crucial role in maintaining cardiovascular health and function, as well as enhancing athletic performance. This review demonstrates that AT-BFR can improve aerobic capacity and muscle performance at low exercise intensities. This approach is particularly beneficial for groups that cannot tolerate high-intensity exercise, such as older adults, individuals undergoing rehabilitation, and endurance athletes during the competitive season. AT-BFR offers a flexible and safe alternative to high-intensity training, allowing for significant improvements in aerobic capacity and strength with minimal risk of overtraining. Its effectiveness is maintained across different training durations and frequencies, with even short-term (2–4 weeks) training showing positive outcomes. However, further research is needed to refine optimal protocols and expand its applicability across different populations and training goals.

## Data Availability

The original contributions presented in the study are included in the article/Supplementary Material, further inquiries can be directed to the corresponding authors.
